# Evaluating sonographic response and remission in inflammatory bowel disease: aligning intestinal ultrasound assessment with STRIDE-II timelines

**DOI:** 10.1093/ecco-jcc/jjag132

**Published:** 2026-09-24

**Authors:** Ashish R Srinivasan, Shintaro Sagami, Andrew Nguyen, Rebecca L Smith, Abhinav Vasudevan, Stuart A Taylor

**Affiliations:** Department of Gastroenterology, Austin Health, Heidelberg, VIC, Australia; Department of Medicine, Austin Academic Centre, University of Melbourne, Melbourne, Australia; Eastern Health Clinical School, Monash University, Melbourne, Australia; Department of Gastroenterology, Eastern Health, Box Hill, VIC, Australia; Center for Advanced IBD Research and Treatment, Kitasato University Kitasato Institute Hospital, Minato, Japan; Department of Gastroenterology, Eastern Health, Box Hill, VIC, Australia; Department of Gastroenterology, Alfred Health, Prahran, VIC, Australia; Eastern Health Clinical School, Monash University, Melbourne, Australia; Department of Gastroenterology, Eastern Health, Box Hill, VIC, Australia; Centre For Medical Imaging, University College London, London, United Kingdom

**Keywords:** intestinal ultrasound, Crohn’s disease, ulcerative colitis, treat-to-target, disease monitoring, treatment response, transmural healing, inflammatory bowel disease, sonographic response, sonographic remission

## Abstract

**Background and Aims:**

The diagnostic accuracy of intestinal ultrasound (IUS) is established; however, guidance on the timing and interpretation of sonographic response and remission in inflammatory bowel disease (IBD) remains limited. This review synthesizes current evidence to define expected response and remission kinetics in Crohn’s disease (CD) and ulcerative colitis (UC) across STRIDE-II aligned reassessment timepoints.

**Methods:**

A structured narrative review was undertaken of studies published up to August 31, 2025 evaluating longitudinal IUS changes following initiation of medical therapy in patients with CD and UC. Outcomes were synthesized across early (<3 months), intermediate (3-6 months), and longer-term (6-12 months) reassessment intervals aligned with STRIDE-II timelines.

**Results:**

In CD, sonographic response was reported in approximately 51%-66% of patients by 12 weeks, while remission was reported in 14%-37%. At 3-6 months, response was reported in approximately 36%-38%, while remission was reported in 12%-38%. Longer-term response and remission were reported in approximately 36%-57% and 24%-42%, respectively, at 48-56 weeks. In UC, response was reported in 57%-77% and remission in 45%-64% by weeks 8-12 in selected cohorts. However, traditional IUS response and remission estimates beyond this early treatment phase remain limited in UC. Direct comparisons between studies was limited by heterogeneity in outcome definitions, reassessment timepoints, therapy class, and disease characteristics.

**Conclusion:**

IUS is a practical, non-invasive tool for monitoring therapeutic response in IBD. The optimal timing of reassessment is likely to vary according to therapy-specific time to response, IBD subtype, disease phenotype, and bowel segment. Standardization of definitions and reassessment timelines remain central to integrating IUS into treat-to-target models of IBD care.

## Graphical abstract

**Graphical Abstract:**
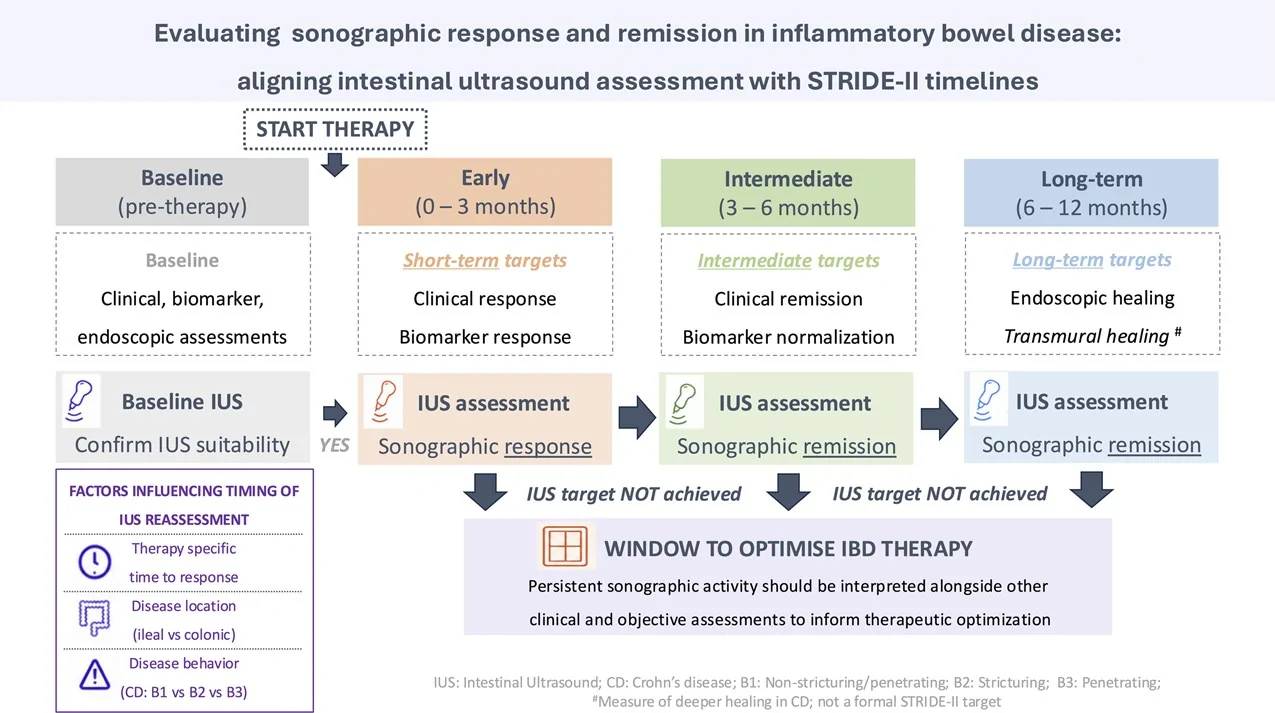

## 1. Introduction

Intestinal ultrasound (IUS) is now embedded in routine inflammatory bowel disease (IBD) care, with established high diagnostic accuracy for detecting active enteric inflammation in both Crohn’s disease (CD) and ulcerative colitis (UC) when performed by trained practitioners.^1–4^ As a safe, noninvasive, cost-effective, and sustainable imaging modality that is preferred by patients, and can be acceptably repeated at short intervals, IUS is well suited to monitoring response to IBD therapy.^4–6^ However, despite its widespread adoption, its role in longitudinal disease monitoring and treat-to-target decision-making remains less clearly defined. A key limitation relates to heterogeneity in definitions of sonographic response and remission, including variability in bowel wall thickness (BWT) thresholds, inconsistent incorporation of color Doppler signal (CDS), and bowel wall stratification (BWS) parameters, and lack of consensus regarding the optimal timing of reassessment following therapy initiation.^7–9^ This was exemplified in a recent systematic review of 51 studies, which highlighted substantial variability in both outcome definitions and reassessment intervals pertaining to IUS.^9^

An international expert consensus has since proposed standardized definitions of sonographic response and remission, including suggested timepoints for sonographic reassessment in clinical trials.^10^ However, how these definitions should be clinically applied within the STRIDE-II treat-to-target framework, outside of clinical trials, remains to be clarified. STRIDE-II defines staged therapeutic targets across short-term (clinical response), intermediate (clinical remission; biomarker normalization), and long-term (mucosal healing) horizons (Table 1).^11^ Despite increasing use of IUS, clear guidance on how sonographic findings align with these treatment phases in routine practice, particularly the timing and magnitude of response and remission, remains poorly defined. This review aims to synthesize current evidence to provide clinicians with practical guidance on the timing and kinetics of sonographic response and remission in CD and UC, aligned with early, intermediate, and long-term timepoints espoused by the STRIDE-II framework.

**Table 1.** Treatment targets endorsed by the Selecting Therapeutic Targets in Inflammatory Bowel Disease-II guidelines in adult Crohn’s disease and ulcerative colitis.

Treatment target	Timepoint	Definition(s)
**Crohn’s disease**		
**Clinical response**	Early	PRO2: ≥50% reduction from baseline in abdominal pain and stool frequency
**Clinical remission**	Intermediate	PRO2: abdominal pain ≤1 and stool frequency ≤3; HBI < 5
**Quality of life and disability**	Long-term	Absence of disability and normalized health-related quality of life
**Biomarker normalization**	Intermediate	FCP < 250 μg/g; CRP < upper threshold of normal range
**Endoscopic response**	Early/Intermediate	> 50% decrease from baseline in SES-CD or CDEIS
**Endoscopic healing/remission**	Long-term	SES-CD ≤ 2 or CDEIS < 3 and absence of ulceration
**Transmural healing^a^**	Long-term	Resolution of transmural inflammation as assessed by MRE, CTE, or IUS
**Ulcerative colitis**		
**Clinical response**	Early	PRO2: ≥50% reduction from baseline in Rectal Bleeding and Stool Frequency
**Clinical remission**	Intermediate	PRO2: rectal bleeding = 0 and stool frequency = 0; partial Mayo (< 3 and no score > 1)
**Quality of life and disability**	Long-term	Absence of disability and normalized health-related quality of life
**Biomarker normalization**	Intermediate	FCP < 250 μg/g; CRP < upper threshold of normal range;
**Endoscopic healing/remission**	Long-term	MES 0; UCEIS ≤ 1
**Histological remission^a^**	Long-term	Absence of active microscopic inflammation on biopsy

a Adjunctive measure of deeper healing beyond endoscopic remission; not a formal STRIDE-II treatment target.

Abbreviations: STRIDE-II, Selecting Therapeutic Targets in Inflammatory Bowel Disease-II; SES-CD, Simple Endoscopic Score for Crohn’s Disease; CDEIS, Crohn’s disease endoscopic index of severity; FCP, fecal calprotectin; CRP, C-reactive protein; MRE, magnetic resonance enterography; CTE, computed tomography enterography; IUS, intestinal ultrasound; MES, Mayo Endoscopic Score; HBI, Harvey Bradshaw Index; PRO, patient reported outcome.

## 2. Methods

A structured search of MEDLINE (via PubMed) and EMBASE was undertaken from database inception to August 31, 2025 using terms relating to IBD and IUS. The search strategy combined controlled and free text terms relating to IBD and IUS, including: (IBD OR inflammatory bowel disease OR ulcerative colitis OR UC OR Crohn’s disease) AND (IUS OR intestinal ultrasound OR gastrointestinal ultrasound OR GIUS) AND (remission OR response). Prospective and retrospective studies in adult patients with CD and UC reporting longitudinal changes in transabdominal IUS parameters following medical therapy were included, provided they specified defined post-treatment timepoints or allowed inference regarding the timing of sonographic response and/or remission.

In view of anticipated heterogeneity in study design, disease subtype, disease location, therapeutic drug class, reassessment intervals, sonographic definitions, and reported endpoints, this review was designed as a structured narrative synthesis rather than a formal systematic review or meta-analysis. Studies that reported serial IUS assessment following initiation of medical therapy for CD or UC were prioritized when deriving reported response and remission ranges across STRIDE-II aligned reassessment intervals. Studies reporting only score derivation, cross-sectional validation, longitudinal changes in individual IUS parameters, or composite activity scores were discussed narratively. Findings were synthesized narratively, grouped by CD and UC, and aligned with STRIDE-II time horizons (early <3 months; intermediate 3-6 months; long-term 6-12 months). Where appropriate, response and remission ranges represent descriptive estimates derived from reported outcomes, rather than statistically pooled estimates. These ranges were intended to provide pragmatic clinical benchmarks for interpreting longitudinal IUS response and remission, and should be interpreted in the context of substantial heterogeneity across available studies. For consistency, the terms sonographic response and sonographic remission are used throughout this review. Where individual studies reported IUS-assessed transmural healing, this was considered within sonographic remission, recognizing that definitions varied across studies and commonly incorporated normalization of BWT, CDS, and other inflammatory ultrasound features.

## 3. Sonographic parameters used to evaluate disease activity in CD and UC

IUS allows assessment of transmural inflammation and can be used to reliably differentiate between active and inactive CD and UC.^29^ Assessment typically focuses on the most affected bowel segment and centers on BWT, CDS, BWS, mesenteric inflammatory fat (i-fat), and the presence of disease-related complications such as strictures, fistulas, and abscesses in CD.^2^^,^^30^^,^^31^

BWT is the most reproducible and clinically informative IUS parameter for disease assessment and monitoring.^29^ A BWT of 3.0 mm or less is generally accepted as consistent with quiescent disease, with consensus recommendation that BWT be evaluated in longitudinal and transverse planes.^8^ Increased BWT correlates strongly with clinical, biochemical, and endoscopic activity in both UC and CD, and remains a consistent finding across all cross-sectional imaging modalities.^32–34^ Reduction in BWT following treatment initiation is consistently associated with therapeutic response, while persistently elevated or markedly increased BWT identifies patients at high risk of treatment failure.^35–37^

CDS reflects bowel wall hyperemia and is a sensitive marker of active inflammation.^38^ Abnormal CDS strongly predicts endoscopic disease activity, with increasing CDS intensity correlating with more severe inflammation.^39^ Failure of CDS to normalize after treatment is associated with poorer clinical outcomes, including steroid dependence and need for surgery.^40^ Loss of BWS has been suggested to reflect deeper transmural inflammatory involvement, frequently associated with ulceration, particularly in CD.^41^ Similarly, the presence of i-fat has been associated with more aggressive disease phenotypes, and has consequently been associated with lower rates of clinical, endoscopic, and transmural remission, particularly in stricturing disease.^42^^,^^43^

## 4. IUS disease activity scores

**Figure 1.**
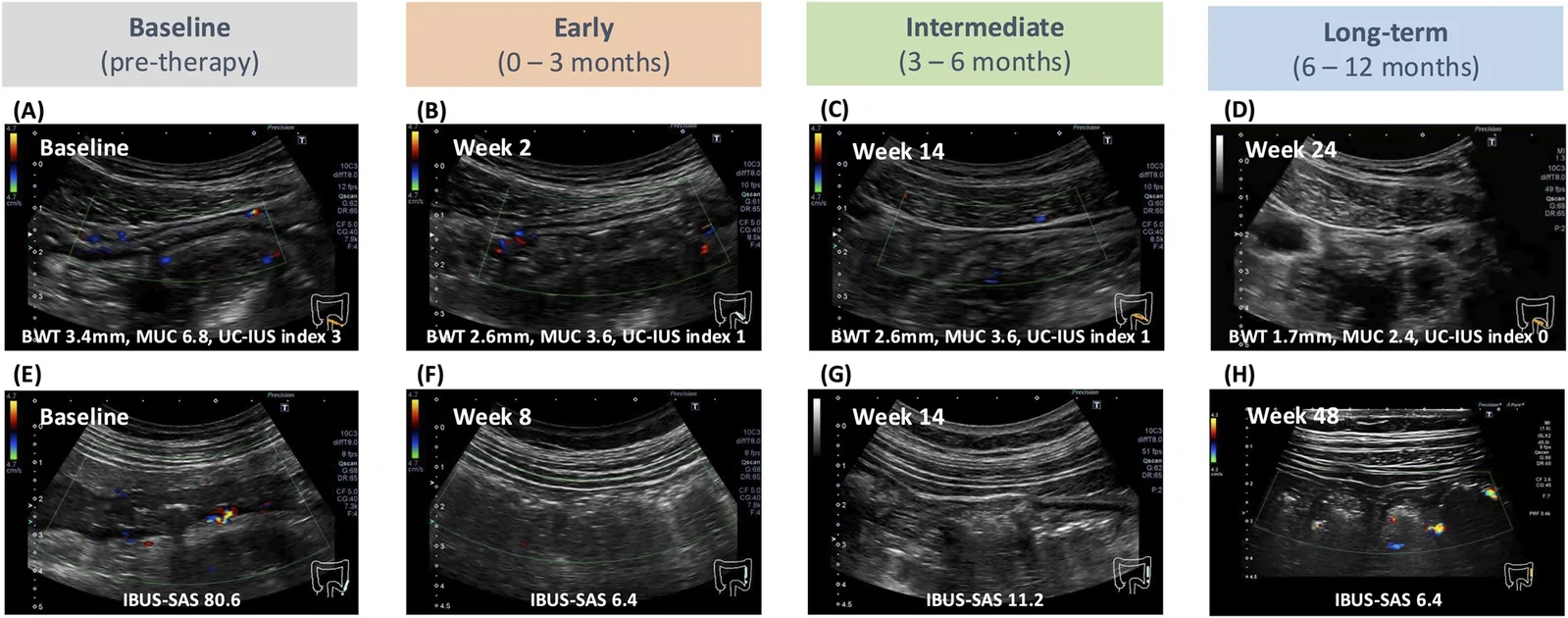
Representative intestinal ultrasound images demonstrating longitudinal changes after treatment initiation in ulcerative colitis and Crohn’s disease. (A-D) Serial images from a patient with left-sided ulcerative colitis treated with upadacitinib, showing the sigmoid colon at baseline (A) and at 2 weeks (B), 14 weeks (C), and 24 weeks (D) after treatment initiation. (E-H) Serial images from a patient with ileocolonic Crohn’s disease treated with upadacitinib, showing the descending colon at baseline (E) and at 8 weeks (F), 14 weeks (G), and 48 weeks (H) after treatment initiation. Abbreviations: BWT, bowel wall thickness; MUC, Milan Ultrasound Criteria; UC-IUS index, ulcerative colitis intestinal ultrasound index; IBUS-SAS, International Bowel Ultrasound Segmental Activity Score.

Although ileo-colonoscopy currently remains the reference standard for assessing disease activity in CD and UC, composite IUS scores offer a method of standardizing sonographic assessment and stratifying sonographic disease activity.^44–46^ By integrating core sonographic parameters, these indices improve reproducibility, facilitate sonographic alignment with clinically meaningful IBD outcomes, and support treat-to-target strategies across both clinical trials and clinical practice (Figure 1).^2^^,^^8^ Multiple IUS-based activity scores have been proposed; however, many were derived from small cohorts, lack robust external validation, and have limited data regarding responsiveness or prognostic performance.^3^^,^^8^ In addition, heterogeneity in score construction and outcome thresholds restricts direct comparison between indices. In CD, the Bowel Ultrasound Score (BUSS) and International Bowel Ultrasound Segmental Activity Score (IBUS-SAS) are the most extensively studied and validated IUS indices, while the Milan Ultrasound Criteria (MUC) score remains the most extensively validated and studied score in UC.^25^^,^^26^^,^^47–53^

## 5. Evaluating sonographic outcomes in clinical practice

As IUS becomes increasingly integrated into treat-to-target IBD care, clear definitions of sonographic response and remission, including their application across defined timepoints following therapy initiation, have become essential. Historically, heterogeneity in response and remission criteria, score complexity, and a lack of consensus regarding optimal reassessment intervals have limited the interpretation and implementation of IUS in both clinical practice and clinical trials.^9^ However, recent international consensus statements have substantially advanced standardization in this area (Table 2).^2^

**Table 2.** Defining sonographic response and remission in Crohn’s disease and ulcerative colitis.

Outcome	Definition(s)^a^
**Crohn’s disease**	
**Sonographic response**	Reduction in inflammatory activity compared with baseline, defined by** ≥ 25% reduction in BWT**, either alone or with** ≥ 1 grade reduction in CDS ** and/or improvement in another IUS inflammatory parameter
**Sonographic remission**	Normalization of inflammatory IUS parameters in previously affected segments, typically defined by **BWT ≤ 3 mm** with absent or minimal vascularity **(CDS 0-1)**. Some definitions also require normalization of BWS, inflammatory fat and other IUS parameters
**Ulcerative colitis**	
**Sonographic response**	Reduction in colonic inflammatory activity compared with baseline, defined by** ≥ 25% reduction in BWT**, either alone or with** ≥ 1 grade reduction in CDS**
**Sonographic remission**	Normalization of colonic inflammatory activity in previously affected segments, generally defined by **normalization of colonic BWT** with absent or minimal vascularity **(CDS 0-1)**. The optimal BWT threshold, particularly in the sigmoid colon, remains uncertain.

a Definitions reflect recent international consensus recommendations for conventional IUS parameter-based endpoints.

Abbreviations: BWT, bowel wall thickness; CDS, color Doppler signal; BWS, bowel wall stratification; IUS, intestinal ultrasound.

Sonographic response is most commonly defined by a meaningful reduction in BWT (≥25% or ≥2 mm), with or without parallel improvement in CDS.^10^ Other definitions of response have also been proposed in prospective cohort studies, and typically combine a ≥25% reduction in BWT with improvement in CDS. Normalization of all sonographic parameters represents the most commonly applied definition of remission or transmural healing on IUS, although there remains debate around the optimal definition of transmural outcomes, which have been designated an adjunctive treatment target in current CD guidelines.^54^^,^^55^ This was also reflected in the recent ECCO-ESGAR-ESP-IBUS guidelines, which position IUS as a first-line, noninvasive tool for CD monitoring and support transmural healing as an important aspirational target alongside mucosal healing.^56^

**Figure 2.**
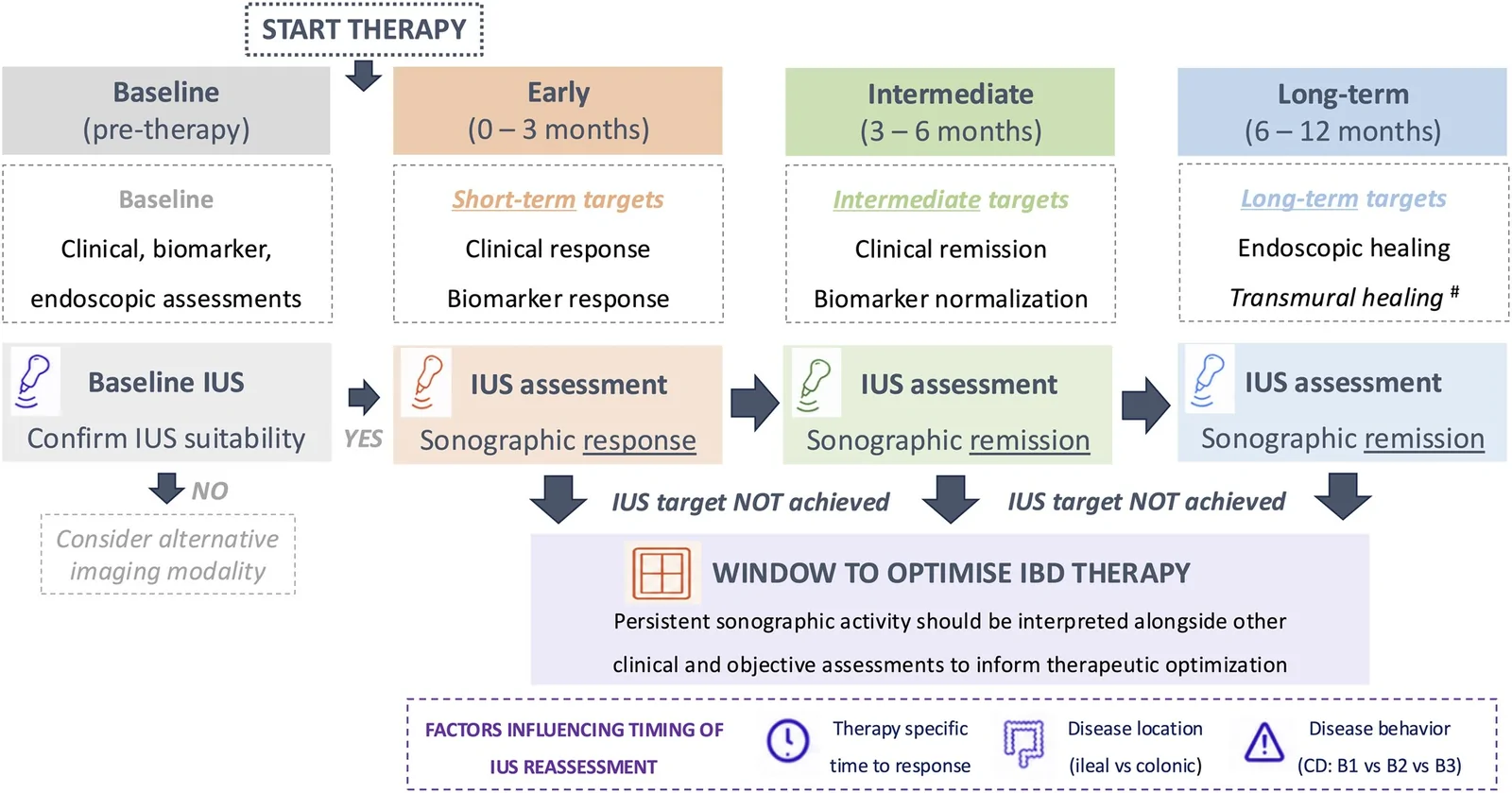
Proposed framework for interpreting IUS response and remission across STRIDE-II aligned reassessment intervals in Crohn’s disease and ulcerative colitis. Abbreviations: IUS, intestinal ultrasound; CD, Crohn’s disease; B1, non-stricturing/penetrating; B2, stricturing; B3, penetrating; #Transmural healing is recognised as an adjunctive measure of deeper healing in CD, rather than as a formal STRIDE-II treatment target.

Definitions of sonographic response and remission must be interpreted in the context of when reassessment is performed. The same sonographic findings may have different implications based on whether they were undertaken at early, intermediate, and longer-term timepoints following IBD therapy initiation. Moreover, factors such as disease location, drug kinetics, disease severity, and disease-related complications may have direct implications for defining reassessment windows and interpreting sonographic outcomes. A proposed framework for the timing and interpretation of IUS reassessment across STRIDE-II treatment phases in CD and UC is summarized in Figure 2.

## 6. Sonographic response and remission in CD

Longitudinal CD cohorts provide the most mature evidence base for IUS-based treatment monitoring in IBD. Across available studies, sonographic response is generally detectable within the first 12 weeks of therapy, while sonographic remission is observed less frequently during this early reassessment window than at later timepoints. The following sections summarize sonographic response and remission in CD across early, intermediate and longer-term intervals aligned with STRIDE-II timelines, with key supporting data summarized in Tables 3 and 4.

**Table 3.** Summary of expected sonographic response and remission across STRIDE-II aligned reassessment intervals following IBD therapy initiation.

STRIDE-II Time horizon	Crohn’s disease^a^	Ulcerative colitis^a^
**Early** **(<3 months)**	**Response:** ∼51-66% by week 12^12^^,^^13^; earlier response at weeks 4-8 is less frequent, reported in 26% in one UST cohort^14^ **Remission/TMH**: ∼14%-37% by week 12^12^^,^^13^^,^^15^; earlier remission at weeks 4-8 was uncommon, reported in 1.8%-6.3% in one UST cohort^14^ Early IUS reassessment is best used to assess sonographic response, as remission/TMH occurs less frequently and is better assessed at later timepoints.	**Response:** ∼57%-77% by weeks 8-12 in selected cohorts^13^^,^^16^; BWT reduction is detectable within 2-8 weeks although response definitions vary^16^^,^^17^ **Remission/TMH:** ∼45%-64% by 8-12 weeks in selected cohorts^13^^,^^18^ **ASUC signal:** early BWT reduction at 48 h predicts corticosteroid response and intervention/colectomy risk^19^^,^^20^
**Intermediate** **(3-6 months)**	**Response:** ∼36%-38% at 3-6 months^14^^,^^21^ **Remission/TMH:** ∼12%-38% at 3-6 months^14^^,^^21–23^ Remission in this timeframe is associated with more favorable outcomes^15^^,^^22^^,^^24^	**Response:** Sustained BWT improvement has been described, but formal response proportions remain limited^16^^,^^17^ **Remission/TMH:** ∼81% at week 16 in one UPA cohort^18^ Persistent sonographic activity predicts relapse, corticosteroid use, escalation, and hospitalization^25^^,^^26^
**Longer-term (6-12 months)**	**Response:** ∼36-57% at 48-54 weeks^12^^,^^14^^,^^21^ **Remission/TMH:** ∼24%-42% at 48-56 weeks^12^^,^^14^^,^^15^^,^^21^^,^^22^^,^^27^ Persistent ileal thickening during maintenance therapy is associated with less favorable outcomes^28^	**Response/Remission:** insufficient eligible IUS data to derive a robust 6-12-month estimate Longitudinal UC data remain less mature than CD.

a Values represent descriptive study-level proportions rather than pooled estimates. Definitions of sonographic response and remission/TMH varied across studies, including BWT/CDS thresholds, bowel segments assessed, therapeutic drug class, and reassessment intervals.

Abbreviations: ASUC, acute severe ulcerative colitis; BWT, bowel wall thickness; CD, Crohn’s disease; CDS, color Doppler signal; IUS, intestinal ultrasound; TMH, transmural healing; UC, ulcerative colitis; UPA, upadacitinib; UST, ustekinumab.

**Table 4.** Longitudinal transabdominal IUS studies contributing to sonographic response and remission estimates following IBD therapy initiation or treatment change.

Study (year, geography)	Disease subtype (IBD therapy)	Sonographic reassessment interval	Sonographic response**^a^**	Sonographic remission/TMH**^a^**
**Ripollés et al.^1^ ^2^** **(2016, Spain)**	CD (IFX, ADA)	Baseline 12 weeks 52 weeks	**Definition:** BWT reduction ≥ 2 mm, CDS reduction by ≥ 1 grade, decreased enhancement ≥ 20%, with or without resolution of complications **Response:** 51.0% at 12 weeks; 57.0% at 52 weeks.	**Definition:** BWT ≤ 3 mm, absent CDS and no complications **Remission/TMH:** 13.7% at 12 weeks; 30.0% at 52 weeks.
**Paredes et al.^15^** **(2019, Spain)**	CD (IFX, ADA)	Baseline 12 weeks 12 months	**Definition:** BWT decrease >2 mm and CDS reduction by ≥ 1 grade **Response:** 18.2% by 12 months.	**Definition:** BWT ≤ 3 mm and CDS ≤ 1 **Remission/TMH:** 21.2% at 12 weeks; 42.4% at 12 months.
**Kucharzik et al.^14^** **(2023, Germany)**	CD (UST)	Baseline 4, 8 weeks 16 weeks 48 weeks	**Definition:** ≥25% BWT reduction **Response:** 25.5% at week 4; 26.6% at week 8; 35.8% at week 16; 46.3% at week 48.	**Definition:** Normalization of BWT, CDS, BWS, and i-fat **Remission/TMH:** 1.8% at week 4; 6.3% at week 8; 11.9% at week 16; 24.1% at week 48
**Madsen et al.^22^** **(2025, Denmark)**	CD (mixed therapies)	Baseline 3 months 6 months 9 months 12 months	**Response:** Not formally defined.	**Definition:** BWT ≤ 3 mm and absent CDS **Remission/TMH:** 38.0% at 3 months; 41.0% at 12 months.
**Calabrese et al.^21^** **(2022, Italy)**	CD (IFX, ADA, UST, VDZ)	Baseline 3 months 6 months 12 months	**Definition:** BWT reduction >1 mm or normalization, reduced disease length, improved CDS, no worsening of other sonographic parameters and no fistula development; **Improved lesions:** 36.7% at 3 months; 38.0% at 6 months; 36% at 12 months.	**Definition:** Normalization of all sonographic parameters; **Remission/TMH:** 16.4% at 3 months; 24.5% at 6 months; 27.5% at 12 months.
**Huang et al.^27^** **(2024, China)**	CD (IFX)	Baseline 14-26 weeks 44-56 weeks	**Response**: Not formally defined.	**Definition:** BWT ≤ 3.0 mm, preserved BWS, CDS 0-1, and absence of i-fat in most affected segment at baseline **Remission/TMH:** 38.0% at 44-56 weeks.
**Ma et al.^23^** **(2021, China)**	CD (mixed therapies)	Baseline 6 months	**Response**: Not formally defined.	**Definition:** BWT ≤ 3 mm, absent CDS, normal BWS, no i-fat, and no complications **Remission/TMH:** 32.0% at 6 months.
**Helwig et al.^13^** **(2022, Germany)**	CD cohort from mixed IBD post-hoc analysis (mixed therapies)	12 weeks	**Definition:** ≥25% BWT reduction **Response:** 65.6% in the CD cohort at week 12.	**Definition:** Multiple transmural healing definitions evaluated, including BWT normalization with CDS normalization, with or without preserved BWS **Remission/TMH:** 23.9-37.2% in the CD cohort at week 12, depending on definition.
**Helwig et al.^13^** **(2022, Germany)**	UC cohort from mixed IBD post-hoc analysis (mixed therapies)	12 weeks	**Definition:** ≥25% BWT reduction **Response:** 76.6% in the UC cohort at week 12.	**Definition:** Multiple TMH definitions evaluated, including BWT normalization with CDS normalization, with or without preserved BWS **Remission/TMH:** 45.0%-61.4% in the UC cohort at week 12, depending on definition.
**Ollech et al.^16^** **(2024, Israel)**	UC (TOFA)	Baseline 8 weeks	**Definition:** BWT ≤ 3 mm and CDS reduction by ≥ 1 grade were assessed as early sonographic improvement parameters **Improvement:** BWT ≤ 3 mm in 18.2%; CDS reduction by ≥ 1 grade in 57.1% at week 8.	**–**
**Gilmore et al.^18^** **(2024, Australia)**	UC (UPA)	Baseline 8 weeks 16 weeks	**Response:** Not formally defined.	**Definition:** BWT <3 mm in all segments and CDS 0 **Remission/TMH:** 64.0% at week 8; 81.0% at week 16 among patients with complete IUS data.

a This table summarizes longitudinal transabdominal IUS studies reporting conventional sonographic response and/or sonographic remission/TMH at defined reassessment timepoints following IBD therapy initiation or modification. These studies provide the key data supporting the STRIDE-II-aligned summary in Table 3.

Abbreviations: ADA, adalimumab; BWS, bowel wall stratification; BWT, bowel wall thickness; CD, Crohn’s disease; CDS, color Doppler signal; IBD, inflammatory bowel disease; IFX, infliximab; i-fat, inflammatory fat; IUS, intestinal ultrasound; TMH, transmural healing; TOFA, tofacitinib; UC, ulcerative colitis; UPA, upadacitinib; UST, ustekinumab; VDZ, vedolizumab.

### 6.1. Early (<3 months)

Within the first 12 weeks of therapy, IUS functions primarily as an objective marker of treatment response rather than remission, aligning with STRIDE-II short-term reassessment intervals. Across longitudinal CD cohorts, sonographic response is reported in approximately 51%-66% by week 12.^12^^,^^13^ In the STARDUST IUS substudy, response defined as a ≥25% reduction in BWT was detectable as early as week 4 following ustekinumab induction, but remained modest at weeks 4 and 8 (25.5% and 26.6%, respectively). Remission was uncommon at these very early timepoints too, occurring in 1.8% and 6.3% at weeks 4 and 8, respectively. Early IUS reassessments should therefore focus on assessments of sonographic response rather than remission, acknowledging that remission occurs more frequently at later timepoints. Similarly, early BWT reduction following anti-tumor necrosis factor (anti-TNF) initiation has been associated with subsequent endoscopic response. Nevertheless, sonographic remission tends to occur less frequently than response across early timepoints, occurring in approximately 14%-37% by week 12.^12–15^

The TRUST CD cohort further supports the value of early reassessment, with anti-TNF-treated patients demonstrating significant sonographic improvement by week 12, including reductions in BWT, decreased CDS, and improvement in composite IUS activity parameters.^57^ Although the original TRUST CD study primarily reported change in sonographic parameters rather than traditional response or remission proportions, a subsequent post-hoc analysis applying standardized definitions reported transmural response, defined by ≥25% BWT reduction, in 65.6% of CD patients by week 12, with remission occurring less frequently (23.9%-37.2%) depending on the definition applied.^13^

### 6.2. Intermediate (3-6 months)

The 3-6-month interval corresponds to STRIDE-II intermediate reassessment intervals, and represents a clinically meaningful sonographic reassessment window during which clinical remission and biomarker normalization are both expected to occur, and is supported by recent ECCO-ESGAR-IBUS guidance.^11^^,^^56^ Across eligible longitudinal CD studies, sonographic response was reported in approximately 36%-38% at 3-6 months, while remission was reported in approximately 12%-38%.^14^^,^^21–23^ Evidence consistently demonstrates that IUS findings around 12 weeks predict longer-term outcomes. In a prospective cohort of 93 patients commencing biologic therapy, week 12 BUSS and IBUS-SAS were associated with subsequent endoscopic and transmural remission, supporting the prognostic value of IUS reassessment at intermediate timepoints.^58^ The Copenhagen IBD Cohort Study, which included patients with newly diagnosed CD, documented that transmural remission (BWT <3 mm with absent CDS) of all segments was achieved in 54 of 141 (38%) patients at 3 months, with more frequent normalization of colonic (L2, 52%) relative to ileal (L1, 33%) segments.^22^ Moreover, patients who achieved transmural remission at 3 months were more likely to achieve steroid-free clinical remission at 3 (83 vs 64%, *P* < .01), 6 (72 vs 45%, *P* < .01), 9 (88 vs 56%, *P* = .01), and 12 (76 vs 49%, *P* = .04) months, indicating that those who achieve transmural remission at this timepoint tend to have a more favorable clinical course.^22^ Similarly, those who achieved transmural remission at 3 months were observed to require less frequent treatment escalation (26 vs 53%, *P* = .003).^22^ Notably, only 54% of CD patients who were in clinical remission at 3 months achieved concurrent transmural remission, illuminating a significant burden of subclinical sonographic disease.

Reduction in BWT alone also has predictive value across intermediate timepoints, with a decrease of approximately 29% between 12 and 34 weeks following initiation of anti-TNF therapy suggested to predict subsequent endoscopic remission (odds ratio [OR]: 37.50, 95% confidence interval [CI]: 2.77-507.48, *P* = .006).^59^ Notably, a BWT cutoff of 3.2 mm was shown to strongly reflect endoscopic remission (OR: 39.42, 95% CI: 7.67-202.57, *P* < .0001).^59^ Another multi-center study of 188 patients with CD treated with various biologics reported transmural healing rates of 16.4%, 24.5%, and 27.5% at 3, 6, and 12 months, respectively.^21^

### 6.3. Longer-term (6-12 months)

Beyond 6 months, corresponding to STRIDE-II long-term targets such as endoscopic healing, IUS provides important prognostic information. At 12 months, transmural remission is observed in approximately 24%-42% of patients across longitudinal cohorts, with those achieving transmural healing experiencing significantly lower rates of corticosteroid use, hospitalization, and surgery.^12^^,^^14^^,^^15^^,^^21^^,^^22^^,^^27^ A substantial proportion of patients who achieve clinical remission continue to demonstrate persistent sonographic activity, particularly in ileal segments.^24^ In a cohort of 202 patients with CD in clinical remission, 61% had persistent IUS activity; however, IUS remission was independently associated with improved medication escalation free survival (hazard ratio [HR] 1.94, 95% CI 1.23-3.06) and corticosteroid-free survival (HR 2.41, 95% CI 1.24-4.67).^24^ Similarly, in an anti-TNF-treated cohort assessed at a mean of 18 months, sonographic improvement was associated with fewer adverse outcomes, while complete transmural healing conferred the most favorable prognosis.^60^

Ileal CD may be particularly vulnerable to adverse long-term outcomes when BWT persists. Among patients receiving maintenance infliximab (mean 75 months from induction [range 30-105]), persisting ileal thickening (≥4 mm) was strongly associated with treatment failure (OR 17.6, 95% CI 4.0-76; *P* = .02).^28^ Persistent ileal thickening has also been associated with the development of stricturing complications, and lower anti-TNF trough levels, compared to colonic disease which achieves transmural normalization more frequently.^21^^,^^61^ The STRIDENT trial further demonstrated that improvements in stricturing disease on IUS at 12 months paralleled endoscopic, biomarker, and magnetic resonance imaging (MRI) outcomes.^62^

Collectively, these data support sonographic reassessment at approximately 3 months as clinically meaningful for evaluating response in CD, with assessment at 6-12 months representing a pragmatic timepoint for evaluating remission. Early intermediate response appears to identify patients who are more likely to achieve sustained remission, while a lack of such response may identify patients who may benefit from therapeutic optimization and/or closer monitoring. Over the longer term, transmural healing is emerging as a clinically meaningful target in CD, a view reinforced by recent ECCO-ESGAR-IBUS guidance, although it is not yet formally embedded within STRIDE-II as a formal treatment target.^56^ Further studies are required to define optimal IUS surveillance intervals once transmural remission has been achieved, and to guide management of discordant endoscopic and transmural outcomes.

## 7. Sonographic response and remission in UC

Although UC has not traditionally been viewed as a transmural disease, recent studies have challenged this dogma, suggesting that submucosal bowel wall alterations may persist even when mucosal healing has been achieved.^63^ In UC, early sonographic improvement is frequently observed during induction therapy, with response reported in approximately 57%-77% by weeks 8-12 in selected cohorts, and remission in approximately 45%-64%.^13^^,^^16^^,^^18^ However, available UC data are more heterogeneous than CD data, with many studies reporting BWT reduction, BWT normalization, MUC-based outcomes, or endoscopic correlations rather than formal traditional response and remission proportions. At intermediate timepoints, sustained BWT improvement has been described and remission has been reported in selected small molecule cohorts, but reporting of formal response outcomes remains limited.^16–18^ Similarly, at 6-12 months, available transabdominal IUS data are insufficient to derive a robust descriptive estimate. The following sections evaluate sonographic response in UC across very early, early, intermediate, and longer-term timepoints.

### 7.1. Acute severe UC

Although acute severe ulcerative colitis (ASUC) falls outside conventional STRIDE-based monitoring horizons, it remains an important clinical setting in which very early sonographic reassessment may influence therapeutic decision-making and short-term outcomes. In ASUC, early identification of corticosteroid non-response is critical to guide rescue therapy and avoid delayed colectomy. IUS has emerged as a rapid bedside assessment tool in this hyper-acute context. Ilvemark et al. demonstrated that an IUS performed within 48 h of intravenous corticosteroid initiation can be used to discriminate responders from non-responders.^19^ At 48 ± 24 h, responders and non-responders could be differentiated on the basis of absolute BWT (median 3.1 mm vs 4.9 mm; *P* < .0001), absolute reduction in BWT (−1.9 mm vs −0.2 mm; *P* < .001), and higher relative reduction in BWT (−35.9% vs −4.1%; *P* < .0001).^19^ Notably, a ≤20% reduction in BWT at 48 ± 24 h had a 84.2% sensitivity and 78.4% specificity for non-response (AUROC 0.85), while a >20% reduction was strongly associated with response (OR 22.6, CI [4.2, 201.2]; *P* = .001).^19^ Persistent BWT ≥ 4 mm at 48 ± 24 h has also been documented to predict need for rescue therapy and colectomy (OR 9.5 [95% CI 1.5-186], *P* = .03), while reduction in BWT < 3 mm has been associated with avoidance of colectomy at 1 year.^19^^,^^20^

These very early changes precede formal STRIDE short-term timepoints but illustrate the potential utility of IUS for dynamic decision-making at very early timepoints in severe disease. Smaller ASUC cohorts similarly suggest that admission BWT and early post-admission BWT assessments may help identify patients who are likely to require rescue therapy, although these data remain exploratory.^64^^,^^65^ Collectively, these findings suggest that ASUC represents a distinct but clinically important extension of time sensitive IUS monitoring within IBD care.

### 7.2. Early (<3 months)

The TRUST&UC study demonstrated rapid sonographic improvement, with the proportion of patients with colonic BWT decreasing from 92.3% at baseline to 28.0% by week 2, with documented stability at weeks 6 and 12.^17^ By week 12, >90% of patients with a BWT < 3 mm achieved clinical response.^13^ Transmural response (≥25% BWT reduction) was documented in 76.6% and was strongly associated with clinical remission at 12 weeks (OR 7.91; *P* < .001), although correlations with C-reactive protein (CRP) and fecal calprotectin (FCP) were less consistent.^13^ In TRUST&UC, normalization of BWT in the sigmoid or descending colon was also associated with higher rates of clinical response (*P* < .001).^17^

Several cohorts have confirmed the value of early IUS in UC. A sigmoid BWT ≤ 3.5 mm within 8-26 weeks of initiating therapy has been associated with endoscopic improvement with high accuracy (AUROC 0.96; 92% sensitivity, 87% specificity), as has a reduction in baseline BWT (AUROC 0.81).^55^ Return of colonic haustration at week 6 has also been independently associated with subsequent endoscopic response (OR 13.5; *P* = .007).^55^ In patients receiving upadacitinib induction, 64% achieved IUS remission by week 8, accompanied by significant reductions in BWT and CDS, with concordant clinical, biochemical, and endoscopic improvement by week 16.^18^

Collectively, these data support IUS reassessment around 8 weeks as a pragmatic early timepoint to assess response to therapy in patients with UC. This timepoint would also provide clinicians with a clinical opportunity to optimize UC therapy prior to consideration of more invasive endoscopic reassessment.

### 7.3. Intermediate (3-6 months) and long-term (6-12 months)

At intermediate timepoints, sustained BWT improvement has been described in UC, but reporting of formal response remains limited. In one upadacitinib induction cohort, BWT/CDS-defined remission was reported in as many as 81% at week 16.^18^ Longer-term prognostic studies using MUC or persistent sonographic activity suggest that residual IUS activity is clinically relevant, with associations reported with relapse, corticosteroid exposure, treatment escalation, and hospitalization.^25^^,^^26^ However, these outcomes are not directly comparable with traditional BWT/CDS-defined remission. Beyond 6 months, eligible transabdominal IUS data remain insufficient to derive robust remission estimates in UC.

## 8. International consensus recommendations

A recent international Delphi consensus standardized the use of IUS as an outcome measure in IBD clinical trials, harmonizing definitions of response and remission, and timing of assessment following therapy initiation.^10^ These recommendations align closely with STRIDE-II treat-to-target principles and emphasize that sonographic response is both time- and segment-dependent. In luminal CD, colonic response assessment was supported as early as 4 weeks (75% agreement), with stronger agreement at 8 weeks (81.2%) and 12 weeks (94.1%), and further reassessment at 24 weeks (93.7%) and 48-52 weeks (82.3%).^10^ Terminal ileal assessment was favored later, at 12 weeks (88.2%), 16 weeks (75%), 24 weeks (93.7%), and 48-52 weeks (82.3%). A reduction in BWT of ≥25% or ≥1 mm at 12-16 weeks (86.6%) was considered an appropriate response threshold, and 88.2% of panelists agreed that reassessment timing should be tailored according to therapy-specific time to response.^10^ In UC, response assessment was supported at 8 weeks (80%) and 12-16 weeks (93.7%), while remission assessment was supported at 12-16 weeks (87.5%), 24-36 weeks (81.2%), and 48-52 weeks (94.1%). Collectively, these recommendations support a more standardized, timepoint-driven approach to longitudinal IUS assessment, while recognizing that reassessment timing may need to vary by IBD subtype, bowel segment and therapy-specific time to response.

## 9. Factors influencing timing of IUS reassessment

The timing and magnitude of sonographic response are unlikely to be uniform across different drug therapies, disease phenotypes, and bowel segments. Earlier changes may be expected with corticosteroids, JAK inhibitors, and anti-TNF agents, while more gradual improvement may occur with other therapies. This is illustrated by very early BWT reduction in ASUC after intravenous corticosteroids, early sonographic improvement during upadacitinib induction in UC, and progressive IUS response and remission through week 48 in ustekinumab-treated CD.^14^^,^^18^^,^^19^ These observations reinforce that therapy-specific time to response should inform the timing of IUS reassessment, particularly during the early period immediately following treatment initiation.

Disease location and behavior are also important clinical considerations. In the STARDUST IUS substudy, colonic segments were associated with higher IUS response and transmural remission than terminal ileal segments by week 48 (62.5% vs 39.5% and 50.0% vs 13.2%, respectively), with differences apparent from 8 weeks.^14^ The Copenhagen CD cohort similarly reported more frequent 3-month transmural remission in colonic compared with ileal segments.^22^ Disease severity and complications, including stricturing or penetrating disease behavior, may further influence the completeness and interpretation of sonographic improvement at different timepoints.^43^ Similarly, the optimal approach to IUS reassessment following dose intensification, primary or adjunctive dietary therapy, or treatment de-escalation are not well defined, as most available data thus far focus on *de novo* initiation of medical therapy.

## 10. Integrating IUS with biomarkers, endoscopy, and clinical decision-making

Beyond determining when to reassess sonographic disease activity, an important unresolved question pertains to whether IUS adds incremental clinical value beyond traditional IBD monitoring tools such as symptom-based assessment, CRP, FCP, magnetic resonance enterography (MRE), and endoscopy. Current data support IUS as a modality that complements rather than replaces these established disease monitoring tools.

The STARDUST IUS substudy demonstrated that early IUS response was associated with subsequent endoscopic response, although agreement with later endoscopic or biomarker outcomes was only fair to moderate (κ = 0.21-0.51).^14^ A prospective anti-TNF tight monitoring CD cohort, published after the search period for this review, further supports the prognostic value of early IUS reassessment, with week 14 IBUS-SAS and BWT predicting week 54 endoscopic remission.^66^ This cohort also demonstrated complementary findings between IUS and MRE, with week 54 IUS response associated with MRE response, and early IUS measures predicting later MRE remission. Although these post-search data were not included in the descriptive estimates presented, they support the complementary role of IUS alongside existing monitoring tools.

Collectively, these findings demonstrate favorable associations between sonographic, biochemical, endoscopic, and radiological markers of disease activity, while also highlighting the potential for discordance. Concordant improvement across IUS and biomarkers may provide clinical reassurance and potentially defer invasive reassessment, while discordant results often require more nuanced interpretation, including evaluation of symptom and biomarker trends to guide the need for further investigation. Prospective studies are therefore needed to determine whether IUS-informed treatment decisions improve meaningful clinical outcomes such as corticosteroid exposure, need for hospitalization or surgery, and treatment escalation. This question is currently being evaluated by VECTORS, a phase 4 interventional randomized trial evaluating whether a treatment target incorporating IUS-defined transmural healing improves outcomes compared with clinical and biomarker remission alone in CD.^67^

## 11. Limitations and future directions

Several important limitations should be considered when interpreting the available literature around longitudinal IUS assessment in IBD. Substantial heterogeneity exists across published studies with respect to IBD subtype, therapeutic drug class, bowel segment assessed, reassessment intervals, and sonographic outcome definitions. This heterogeneity limited formal meta-analysis and instead supported a structured narrative approach aimed at providing pragmatic clinical guidance. The response and remission ranges presented in this review should therefore be interpreted as descriptive study-level data rather than statistically pooled estimates. These limitations are particularly relevant when comparing response kinetics across CD and UC, ileal and colonic segments, and drugs with different time to response. In UC, eligible transabdominal IUS data beyond the induction period remain limited. This review focuses primarily on *de novo* initiation of medical therapy, and optimal IUS reassessment windows following escalation or de-escalation of medical therapy remain to be defined.

Although IUS is increasingly used in IBD monitoring, it remains operator dependent and requires structured training to ensure that sonographic parameters are evaluated and interpreted in a clinically useful and reproducible manner. International bowel ultrasound (IBUS) initiatives and recent ECCO-ESGAR-ESP-IBUS recommendations have helped standardize how IUS should be performed, assessed, reported, and interpreted in this context.^56^^,^^68^^,^^69^ Nevertheless, significant variability in regional training pathways and scanning requirements highlight concerns regarding interobserver variability and reporting. Structured reporting templates, longitudinal storage of IUS images, and collaboration between gastroenterologists and radiologists experienced in IUS will be important to support and optimize the integration of IUS into routine IBD care.^70^^,^^71^

## 12. Conclusion

IUS is an accurate, noninvasive, and patient-centric tool that is increasingly integrated into contemporary IBD care. Across CD and UC, reductions in BWT and CDS generally parallel clinical, biochemical, and endoscopic improvement, although the timing and magnitude of sonographic response are influenced by several disease- and therapy-related factors. Recent international consensus efforts have helped standardize definitions of sonographic response and remission; however, guidance on the optimal timing of reassessment remains limited, particularly in relation to therapy-specific time to response, disease severity, and phenotype. By synthesizing available longitudinal data, this review provides pragmatic descriptive data to help clinicians interpret sonographic response and remission across STRIDE-II aligned time horizons, while highlighting important evidence gaps. Given the rapidly evolving literature on IUS in IBD, relevant longitudinal studies continue to emerge following completion of the structured search period for this review and may further refine our understanding. Further prospective studies are needed to determine whether early sonographic response predicts durable remission across IBD subtypes, therapy classes, and disease locations, and whether incorporating IUS into treat-to-target algorithms improves long term objective outcomes. Together, these data will help define how IUS is best integrated into treat-to-target models of IBD care.

## Data Availability

Data sharing is not applicable to this article as no new data were created.

## References

[1] Smith RL , Taylor KM, Friedman AB, Gibson RN, Gibson PR. Systematic review: clinical utility of gastrointestinal ultrasound in the diagnosis, assessment and management of patients with ulcerative colitis. J Crohns Colitis. 2020;14:465-479. 10.1093/ecco-jcc/jjz163 31562739

[2] Ilvemark JFKF , Hansen T, Goodsall TM, et al Defining transabdominal intestinal ultrasound treatment response and remission in inflammatory bowel disease: systematic review and expert consensus statement. J Crohns Colitis. 2022;16:554-580. 10.1093/ecco-jcc/jjab173 PMC9089416 34614172

[3] Goodsall TM , Nguyen TM, Parker CE, et al Systematic review: gastrointestinal ultrasound scoring indices for inflammatory bowel disease. J Crohns Colitis. 2021;15:125-142. 10.1093/ecco-jcc/jjaa129 32614386

[4] Srinivasan A , Wong D, Porwal R, et al Access to intestinal ultrasound influences diagnostic decision-making, reduces healthcare costs and improves sustainability in inflammatory bowel disease. Frontline Gastroenterol. 2026;flgastro-2025-103577. 10.1136/flgastro-2025-103577

[5] Chen L , Ruddick-Collins L, An Y-K, et al Examining attitudes to intestinal ultrasound in inflammatory bowel disease: a national survey of Australian gastroenterologists. Ther Adv Gastroenterol. 2026;19:17562848261432538. 10.1177/17562848261432538 PMC13009771 41883698

[6] Rajagopalan A , Sathananthan D, An Y-K, et al Gastrointestinal ultrasound in inflammatory bowel disease care: Patient perceptions and impact on disease-related knowledge. JGH Open. 2020;4:267-272. 10.1002/jgh3.12268 PMC7144798 32280776

[7] Wilkens R , Novak KL, Maaser C, Panaccione R, Kucharzik T. Relevance of monitoring transmural disease activity in patients with Crohn’s disease: current status and future perspectives. Ther Adv Gastroenterol. 2021;14:17562848211006672. 10.1177/17562848211006672 PMC8053830 33948115

[8] Goodsall TM , Jairath V, Feagan BG, et al Standardisation of intestinal ultrasound scoring in clinical trials for luminal Crohn’s disease. Aliment Pharmacol Ther. 2021;53:873-886. 10.1111/apt.16288 33641221

[9] Allocca M , D’Amico F, Fiorino G, et al Systematic review on definitions of intestinal ultrasound treatment response and remission in inflammatory bowel disease. J Crohns Colitis. 2025;19:jjaf011. 10.1093/ecco-jcc/jjaf011 39825748

[10] Allocca M , Jairath V, Sands BE, et al International consensus on the use of intestinal ultrasound in inflammatory bowel disease trials. J Crohns Colitis. 2025;19:jjaf170. 10.1093/ecco-jcc/jjaf170 40973472

[11] Turner D , Ricciuto A, Lewis A, et al; International Organization for the Study of IBD. STRIDE-II: an update on the Selecting Therapeutic Targets in Inflammatory Bowel Disease (STRIDE) initiative of the International Organization for the Study of IBD (IOIBD): determining therapeutic goals for treat-to-target strategies in IBD. Gastroenterology. 2021;160:1570-1583. 10.1053/j.gastro.2020.12.031 33359090

[12] Ripollés T , Paredes JM, Martínez-Pérez MJ, et al Ultrasonographic changes at 12 weeks of anti-TNF drugs predict 1-year sonographic response and clinical outcome in Crohn’s disease: a multicenter study. Inflamm Bowel Dis. 2016;22:2465-2473. 10.1097/MIB.0000000000000882 27580385

[13] Helwig U , Fischer I, Hammer L, et al Transmural response and transmural healing defined by intestinal ultrasound: new potential therapeutic targets? J Crohns Colitis. 2022;16:57-67. 10.1093/ecco-jcc/jjab106 34185843

[14] Kucharzik T , Wilkens R, D’Agostino M-A, et al; STARDUST Intestinal Ultrasound study group. Early ultrasound response and progressive transmural remission after treatment with ustekinumab in Crohn’s disease. Clin Gastroenterol Hepatol. 2023;21:153-163.e112. 10.1016/j.cgh.2022.05.055 35842121

[15] Paredes JM , Moreno N, Latorre P, et al Clinical impact of sonographic transmural healing after anti-TNF antibody treatment in patients with Crohn’s disease. Dig Dis Sci. 2019;64:2600-2606. 10.1007/s10620-019-05567-w 30874986

[16] Ollech JE , Eran-Banai H, Goren I, et al Tofacitinib is an effective treatment for moderate to severe ulcerative colitis, and intestinal ultrasound can discriminate response from non-response: a pragmatic prospective real-world study. Ann Med. 2024;56:2358183. 10.1080/07853890.2024.2358183 PMC11141311 38813808

[17] Maaser C , Petersen F, Helwig U, et al; German IBD Study Group and TRUST&UC study group. Intestinal ultrasound for monitoring therapeutic response in patients with ulcerative colitis: results from the TRUST&UC study. Gut. 2020;69:1629-1636. 10.1136/gutjnl-2019-319451 PMC7456734 31862811

[18] Gilmore R , Fernandes R, Hartley I, et al Upadacitinib induction is effective and safe in ulcerative colitis patients including those with prior exposure to tofacitinib: a multicenter real-world cohort study. Intest Res. 2025;23:347-357. 10.5217/ir.2024.00127 PMC12332279 39709985

[19] Ilvemark JFKF , Wilkens R, Thielsen P, et al Early intestinal ultrasound predicts intravenous corticosteroid response in hospitalised patients with severe ulcerative colitis. J Crohns Colitis. 2022;16:1725-1734. 10.1093/ecco-jcc/jjac083 35695823

[20] Ilvemark JFKF , Wilkens R, Thielsen P, et al Early intestinal ultrasound in severe ulcerative colitis identifies patients at increased risk of 1-year treatment failure and colectomy. J Crohns Colitis. 10.1093/ecco-jcc/jjae101 2024 38940464

[21] Calabrese E , Rispo A, Zorzi F, et al Ultrasonography tight control and monitoring in Crohn’s disease during different biological therapies: a multicenter study. Clin Gastroenterol Hepatol. 2022;20:e711-e722. 10.1016/j.cgh.2021.03.030 33775896

[22] Madsen GR , Attauabi M, Ilvemark JFKF, et al Intestinal ultrasound findings and their prognostic value in early Crohn’s disease: a Copenhagen IBD cohort study. *Clin Gastroenterol Hepatol*. 2025;23:1398-1407.e6. 10.1016/j.cgh.2024.12.030 40081637

[23] Ma L , Li W, Zhuang N, et al Comparison of transmural healing and mucosal healing as predictors of positive long-term outcomes in Crohn’s disease. Ther Adv Gastroenterol. 2021;14:17562848211016259. 10.1177/17562848211016259 PMC8193655 34178114

[24] Vaughan R , Tjandra D, Patwardhan A, et al Toward transmural healing: Sonographic healing is associated with improved long-term outcomes in patients with Crohn’s disease. Aliment Pharmacol Ther. 2022;56:84-94. 10.1111/apt.16892 PMC9313877 35343603

[25] Maeda M , Sagami S, Tashima M, et al Milan Ultrasound Criteria predict relapse of ulcerative colitis in remission. Inflamm Intest Dis. 2023;8:95-104. 10.1159/000532052 PMC10718580 38098495

[26] Allocca M , Dell’Avalle C, Craviotto V, et al Predictive value of Milan ultrasound criteria in ulcerative colitis: A prospective observational cohort study. United European Gastroenterol J. 2022;10:190-197. 10.1002/ueg2.12206 PMC8911545 35233934

[27] Huang Z , Cheng W, Chao K, et al Baseline and postinduction intestinal ultrasound findings predict long-term transmural and mucosal healing in patients with Crohn’s disease. Inflamm Bowel Dis. 2024;30:1767-1775. 10.1093/ibd/izad251 37889843

[28] Albshesh A , Ungar B, Ben-Horin S, Eliakim R, Kopylov U, Carter D. terminal ileum thickness during maintenance therapy is a predictive marker of the outcome of infliximab therapy in Crohn disease. Inflamm Bowel Dis. 2020;26:1619-1625. 10.1093/ibd/izaa219 32860057

[29] Bryant RV , Friedman AB, Wright EK, et al Gastrointestinal ultrasound in inflammatory bowel disease: an underused resource with potential paradigm-changing application. Gut. 2018;67:973-985. 10.1136/gutjnl-2017-315655 29437914

[30] Nagarajan KV , Bhat N. Intestinal ultrasound in inflammatory bowel disease: New kid on the block. Indian J Gastroenterol. 2024;43:160-171. 10.1007/s12664-023-01468-z 37996771

[31] Castiglione F , Mainenti PP, De Palma GD, et al Noninvasive diagnosis of small bowel Crohn’s disease: direct comparison of bowel sonography and magnetic resonance enterography. Inflamm Bowel Dis. 2013;19:991-998. 10.1097/MIB.0b013e3182802b87 23429465

[32] Antonelli E , Giuliano V, Casella G, et al Ultrasonographic assessment of colonic wall in moderate-severe ulcerative colitis: comparison with endoscopic findings. Dig Liver Dis. 2011;43:703-706. 10.1016/j.dld.2011.02.019 21482208

[33] Takahara M , Hiraoka S, Ohmori M, et al The colon wall thickness measured using transabdominal ultrasonography is useful for detecting mucosal inflammation in ulcerative colitis. Intern Med. 2022;61:2703-2709. 10.2169/internal­medicine.8827-21 PMC9556236 35185047

[34] Dolinger MT , Aronskyy I, Kellar A, et al Determining the accuracy of intestinal ultrasound scores as a prescreening tool in Crohn’s disease clinical trials. Am J Gastroenterol. 2024;119:930-936. 10.14309/ajg.0000000000002632 38131626

[35] Maconi G , Ardizzone S, Parente F, Bianchi Porro G. Ultrasonography in the evaluation of extension, activity, and follow-up of ulcerative colitis. Scand J Gastroenterol. 1999;34:1103-1107. 10.1080/003655299750024904 10582761

[36] Albshesh A , Abend A, Yehuda RM, et al Intestinal ultrasound accurately predicts future therapy failure in Crohn’s disease patients in a biologics-induced remission. Eur J Gastroenterol Hepatol. 2025;37:184-189. 10.1097/MEG.0000000000002883 39514257

[37] Castiglione F , de Sio I, Cozzolino A, et al Bowel wall thickness at abdominal ultrasound and the one-year-risk of surgery in patients with Crohn’s disease. Am J Gastroenterol. 2004;99:1977-1983. 10.1111/j.1572-0241.2004.40267.x 15447760

[38] Sasaki T , Kunisaki R, Kinoshita H, et al Use of color Doppler ultrasonography for evaluating vascularity of small intestinal lesions in Crohn’s disease: correlation with endoscopic and surgical macroscopic findings. Scand J Gastroenterol. 2014;49:295-301. 10.3109/00365521.2013.871744 24344807

[39] Bots S , Nylund K, Gecse K. Intestinal ultrasound to assess disease activity in ulcerative colitis: development of a novel UC-Ultrasound Index. J Crohns Colitis. 2022;16:337. 10.1093/ecco-jcc/jjab148 34389853

[40] Ripollés T , Martínez MJ, Barrachina MM. Crohn’s disease and color Doppler sonography: response to treatment and its relationship with long-term prognosis. J Clin Ultrasound. 2008;36:267-272. 10.1002/jcu.20423 18067121

[41] Kunihiro K , Hata J, Haruma K, Manabe N, Tanaka S, Chayama K. Sonographic detection of longitudinal ulcers in Crohn disease. Scand J Gastroenterol. 2004;39:322-326. 10.1080/00365520310008485 15125463

[42] Cheng W , Huang Z, Qin S, et al Inflammatory mesenteric fat detected by intestinal ultrasound is correlated with poor long-term clinical outcomes in patients with Crohn’s disease. Dig Liver Dis. 2024;56:723-729. 10.1016/j.dld.2023.10.016 38061972

[43] Ma L , He Y, Li W, et al Ultrasound characteristics can predict response to biologics therapy in stricturing Crohn’s disease. Clin Transl Gastroenterol. 2024;15:e00738. 10.14309/ctg.0000000000000738 PMC11346852 38976327

[44] Khanna R , Nelson SA, Feagan BG, et al Endoscopic scoring indices for evaluation of disease activity in Crohn’s disease. Cochrane Database Syst Rev. 2016;2016:CD010642. 10.1002/14651858.CD010642.pub2 PMC7079710 27501379

[45] Lobatón T , Bessissow T, De Hertogh G, et al The Modified Mayo Endoscopic Score (MMES): a new index for the assessment of extension and severity of endoscopic activity in ulcerative colitis patients. J Crohns Colitis. 2015;9:846-852. 10.1093/ecco-jcc/jjv111 26116558

[46] Ikeya K , Hanai H, Sugimoto K, et al The ulcerative colitis endoscopic index of severity more accurately reflects clinical outcomes and long-term prognosis than the mayo endoscopic score. J Crohns Colitis. 2016;10:286-295. 10.1093/ecco-jcc/jjv210 PMC4957474 26581895

[47] Novak KL , Nylund K, Maaser C, et al Expert consensus on optimal acquisition and development of the International Bowel Ultrasound Segmental Activity Score [IBUS-SAS]: a reliability and inter-rater variability study on intestinal ultrasonography in Crohn’s disease. J Crohns Colitis. 2021;15:609-616. 10.1093/ecco-jcc/jjaa216 PMC8023841 33098642

[48] Dragoni G , Gottin M, Innocenti T, et al Correlation of ultrasound scores with endoscopic activity in Crohn’s disease: a prospective exploratory study. J Crohns Colitis. 2023;17:1387-1394. 10.1093/ecco-jcc/jjad068 37023010

[49] Allocca M , Craviotto V, Bonovas S, et al Predictive value of bowel ultrasound in Crohn’s disease: a 12-month prospective study. Clin Gastroenterol Hepatol. 2022;20:e723-e740. 10.1016/j.cgh.2021.04.029 33895360

[50] Allocca M , Craviotto V, Dell’Avalle C, et al Bowel ultrasound score is accurate in assessing response to therapy in patients with Crohn’s disease. Aliment Pharmacol Ther. 2022;55:446-454. 10.1111/apt.16700 34783066

[51] Allocca M , Filippi E, Costantino A, et al Milan ultrasound criteria are accurate in assessing disease activity in ulcerative colitis: external validation. United European Gastroenterol J. 2021;9:438-442. 10.1177/2050640620980203 PMC8259285 33349199

[52] Goodsall TM , Day AS, Andrews JM, Ruszkiewicz A, Ma C, Bryant RV. Composite assessment using intestinal ultrasound and calprotectin is accurate in predicting histological activity in ulcerative colitis: a cohort study. Inflamm Bowel Dis. 2024;30:190-195. 10.1093/ibd/izad043 PMC10834160 36928672

[53] Yuan B , Huang P, Yang M, Tang G, Wang F. Intestinal ultrasound scan predicts corticosteroid failure and colectomy risk in patients with ulcerative colitis. Eur J Gastroenterol Hepatol. 2024;36:884-889. 10.1097/MEG.0000000000002780 38652524

[54] Srinivasan AR. Treat to target in Crohn’s disease: a practical guide for clinicians. World J Gastroenterol. 2024;30:50-69. 10.3748/wjg.v30.i1.50 PMC10823901 38293329

[55] Lo RW , Bhatnagar G, Kutaiba N, Srinivasan AR. Evaluating luminal and post-operative Crohn’s disease activity on magnetic resonance enterography: a review of radiological disease activity scores. World J Gastroenterol. 2025;31:107419. 10.3748/wjg.v31.i26.107419 PMC12264814 40678704

[56] Kucharzik T , Taylor S, Allocca M, et al ECCO-ESGAR-ESP-IBUS guideline on diagnostics and monitoring of patients with inflammatory bowel disease: Part 1: Initial diagnosis, monitoring of known inflammatory bowel disease, detection of complications. J Crohns Colitis. 2025;19:jjaf106. 10.1093/ecco-jcc/jjaf106

[57] Kucharzik T , Wittig BM, Helwig U, et al; TRUST study group. Use of intestinal ultrasound to monitor Crohn’s disease activity. Clin Gastroenterol Hepatol. 2017;15:535-542.e532. 10.1016/j.cgh.2016.10.040 27856365

[58] Allocca M , Dell’Avalle C, Zilli A, et al Ultrasound remission after biologic induction and long-term endoscopic remission in Crohn’s disease: a prospective cohort study. EClinicalMedicine. 2024;71:102559. 10.1016/j.eclinm.2024.102559 PMC10972812 38549587

[59] de Voogd F , Bots S, Gecse K, Gilja OH, D’Haens G, Nylund K. Intestinal ultrasound early on in treatment follow-up predicts endoscopic response to Anti-TNFα treatment in Crohn’s disease. J Crohns Colitis. 2022;16:1598-1608. 10.1093/ecco-jcc/jjac072 PMC9624292 35639823

[60] Zorzi F , Ghosh S, Chiaramonte C, et al Response assessed by ultrasonography as target of biological treatment for Crohn’s disease. Clin Gastroenterol Hepatol. 2020;18:2030-2037. 10.1016/j.cgh.2019.10.042 31866561

[61] Maconi G , Lepore F, Saleh A, et al Factors correlated with transmural healing in patients with Crohn’s disease in long-term clinical remission on anti-TNF medication. Dig Liver Dis. 2024;56:2052-2059. 10.1016/j.dld.2024.05.026 38897858

[62] Schulberg JD , Wright EK, Holt BA, et al Intensive drug therapy versus standard drug therapy for symptomatic intestinal Crohn’s disease strictures (STRIDENT): an open-label, single-centre, randomised controlled trial. Lancet Gastroenterol Hepatol. 2022;7:318-331. 10.1016/S2468-1253(21)00393-9 34890567

[63] Eggermont E , Gecse K, Cleveland NK, et al Ulcerative colitis: moving beyond the mucosal dogma. Lancet Gastroenterol Hepatol. 2026;11:163-174. 10.1016/S2468-1253(25)00263-8 41173014

[64] Smith RL , Taylor KM, Friedman AB, Swaine AP, Gibson DJ, Gibson PR. Early assessment with gastrointestinal ultrasound in patients hospitalised for a flare of ulcerative colitis and predicting the need for salvage therapy: a pilot study. Ultrasound Med Biol. 2021;47:1108-1114. 10.1016/j.ultrasmedbio.2020.12.001 33413967

[65] Gilmore R , Tan WL, Fernandes R, An Y-K, Begun J. Upadacitinib salvage therapy for infliximab-experienced patients with acute severe ulcerative colitis. J Crohns Colitis. 2023;17:2033-2036. 10.1093/ecco-jcc/jjad115 PMC10798861 37422724

[66] Palmela C , Revés J, Frias-Gomes C, et al Use of intestinal ultrasound in a tight monitoring approach in Crohn’s disease: a multicentre prospective study. Clinical Gastroenterology and Hepatology. 10.1016/j.cgh.2026.03.042 2026 41997335

[67] Jairath V , Vuyyuru SK, Zou G, et al Evaluating treatment to a target of transmural healing in patients with moderately to severely active Crohn’s disease: rationale, design and protocol for the randomised controlled VECTORS trial. BMJ Open Gastroenterol. 2026;13:e002088. 10.1136/bmjgast-2025-002088 PMC12927283 41720589

[68] Yanai H , Feakins R, Allocca M, et al ECCO-ESGAR-ESP-IBUS guideline on diagnostics and monitoring of patients with inflammatory bowel disease: part 2: IBD scores and general principles and technical aspects. J Crohns Colitis. 2025;19:jjaf107. 10.1093/ecco-jcc/jjaf107

[69] Kucharzik T , Tielbeek J, Carter D, et al ECCO-ESGAR topical review on optimizing reporting for cross-sectional imaging in inflammatory bowel disease. J Crohns Colitis. 2022;16:523-543. 10.1093/ecco-jcc/jjab180 34628504

[70] Lo R , Ma Z, Chen L, Vasudevan A, Srinivasan A. Structured versus non‐structured reporting of inflammatory bowel disease imaging: a systematic review. JGH Open. 2025;9:e70288. 10.1002/jgh3.70288 PMC12477271 41031089

[71] Srinivasan A , Taylor SA. Invited commentary: imaging in Crohn disease is evolving: why clinician-radiologist collaboration has never been more important. Radiographics. 2026;46:e250261. 10.1148/rg.250261 42207682

